# Stretchable Triboelectric Fiber for Self-powered Kinematic Sensing Textile

**DOI:** 10.1038/srep35153

**Published:** 2016-10-11

**Authors:** Hyeon Jun Sim, Changsoon Choi, Shi Hyeong Kim, Kang Min Kim, Chang Jun Lee, Youn Tae Kim, Xavier Lepró, Ray H. Baughman, Seon Jeong Kim

**Affiliations:** 1 Center for Self-Powered Actuation, Department of Biomedical Engineering, Hanyang University, Seoul 04763, Korea; 2 IT Fusion Technology Research Center and Department of IT Fusion Technology, Chosun University, Gwangju 61452, Korea; 3 The Alan G. MacDiarmid NanoTech Institute, University of Texas at Dallas, Richardson, TX 75083, USA

## Abstract

Stretchable fiber and yarn triboelectric nanogenerator are sought for such applications as wearable sensing system such as cloth communication devices, electronic textiles, and robotic sensory skin. Unfortunately, previously reported triboelectric fiber and yarn are difficult to have stretchable property. We introduce here a new type of stretchable and weavable triboelectric fibers with microdiameter dimensions. The stretchable triboelectric fibers can be reversibly stretched up to 50% in tensile direction while generating voltage output proportional to the applied tensile strain. The reversible distance change induced by the Poisson’s ratio difference between the core fiber (silver-coated nylon/polyurethane) and the shell (wrinkled polyvinylidene fluoride-co-trifluoroethylene/carbon nanotube layer) during tensile deformation is the key working principle for electrical generation. Owing to exceptional structural stability, the stretchable triboelectric fibers show high performance retention after 10,000 times repeated stretching/releasing cycle. Furthermore, the stretchable triboelectric fibers are mechanically strong to be woven into a commercial textile for textile based sensors, which can detect magnitude as well as direction of the motion.

Wearable kinematic sensing systems have attracted considerable attention in the past decade with the growing industry of wearable electronics and ubiquitous healthcare. These sensing systems have a high potential for usage in a wide range of industrial applications such as wearable communication devices, electronic textiles, robotic sensory skin, and biomedical devices^1^,^2^,^3^,^4^,^5^. Some kinematic sensing systems for strain detection are based on resistance^6^,^7^,^8^,^9^,^10^, capacitance^11^,^12^,^13^,^14^, electromagnetic interaction^15^,^16^, piezoelectric^17^,^18^,^19^,^20^ and triboelectric effects^21^,^22^,^23^,^24^,^25^,^26^,^27^,^28^,^29^. Among the various strain sensors, the sensing systems based on the triboelectric effect have been intensively studied as self-powered sensors that operate without an external energy source. Triboelectric-based sensors have unique advantages for their simple design, high energy-converting efficiency, low cost, and high sensitivity^22^,^23^,^24^,^25^,^26^.

Wearable sensor mostly focus on the textile-type sensing system, which can be detected from human motion, and the device can transformed from a 3-dimensional (3D) or 2-dimensional (2D) structure to a 1-dimensional (1D) fiber structure^18^,^19^,^20^. These 1D fibers have a high mechanical degree of freedom and are used as the building blocks of the textile. However, it is an elusive goal to be able to weave a highly stretchable sensing fiber textile. One of the problems is the low elastic property of the human motion-sensing textile that can restrict human motion in daily life, and it is difficult to be applied to areas of that body that are highly deformable (to strain of ~50%), such as fingers, elbows, and knee joints.

## Results

### Preparation of stretchable triboelectric fiber

**Figure 1:**
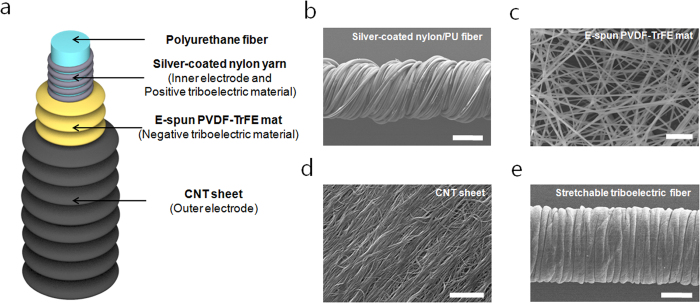
Stretchable triboelectric fiber structure and morphology. (**a**) Schematic diagram of stretchable triboelectric structure. SEM image of (**b**) the silver-coated nylon yarn-wrapped PU fiber (scale bar: 200 μm); (**c**) electrospun PVDF-TrFE fibers (scale bar: 10 μm); (**d**) CNT sheet (scale bar: 2 μm); and (**e**) the final fabricated triboelectric fiber (scale bar: 200 μm).

The stretchable triboelectric fiber (STEF) formed a multilayered core–shell and wrinkle structure (Fig. 1a). To create the STEF, we first designed a new type of stretchable electrode on which silver-coated nylon yarns were wrapped around a polyurethane (PU) fiber (Fig. 1b; Supplementary Fig. S1). The silver-coated nylon yarn consisted of silver-coated nylon 6,6 monofilament 30 μm in diameter which has a high conductivity and electrical stability in deformation^18^,^19^. The silver-coated nylon/PU fiber had an average diameter of 440 μm with a resistance of 10.4 Ohm at the initial state of 10 mm length (Supplementary Fig. S2a). The resistance increased linearly with a strain of 13.3 Ohm at 50%. When the silver-coated nylon/PU fiber was stretched, the PU fiber elongated longitudinally and shrunk in the radial direction. At the same time, a gap was created between the wrapped silver-coated nylon yarn around the PU fiber, resulting a detachment of the adjacent silver-coated nylon yarn at the gap position. Although the electrical pathway of the silver-coated nylon/PU fiber was elongated, the electrical pathway along the silver-coated nylon yarn was constant. As a result, the silver-coated nylon/PU fiber showed stretchable electrode performance.

Secondly, electrospun mats, prepared from polyvinylidene fluoride-co-trifluoroethylene (PVDF-TrFE), were manually wrapped around the silver-coated nylon/PU fiber. The electrospun mats consisted of randomly oriented nanofibers with an average diameter of 750 nm (Fig. 1c). The PVDF-TrFE was chosen for their high negativity in the triboelectric series. We used the electrospinning method, which is a versatile to yield fine-scale fibers, to fabricate high surface roughness of PVDF-TrFE mats for enhanced triboelectric performance^30^,^31^.

Then, carbon nanotube (CNT) sheets were drawn from a forest of chemical vapor deposited multiwalled CNTs and were wrapped around the 180% strained PVDF-TrFE/silver-coated nylon/PU fiber. However, after releasing the CNT/PVDF-TrFE/silver-coated nylon/PU fiber, the length of the fiber increased to about 120% strain. The PVDF-TrFE/CNT shell interrupted the recovery of the PU fiber by forming a wrinkled structure on the surface of the fiber. Despite non-elastic property of PVDF-TrFE, the electrospun mats can absorb large tensile strain (up to 180% in our study) by aligning the randomly oriented nanofibers during first stretching. When the fabrication strain is released, plastically deformed PVDF-TrFE mats get uniform and closely packed wrinkles by recovery force of core fiber (Supplementary Fig. S3). Also, we observed that the more uniform wrinkles were successfully fabricated by wrapping the PVDF-TrFE mats on the core fiber before application of tensile strain. The resistance of 10 mm wrinkled PVDF-TrFE/CNT shell was 5.03 kOhm at the initial state and a constant strain of 50% (Supplementary Fig. S2b). The CNT sheets showed high conductivity and strongly adhered to the PVDF-TrFE by their nanostructure^19^ (Fig. 1d). Also the PVDF-TrFE/CNT shell acted as one body system in deformation. When the strained fiber was released, the PVDF-TrFE/CNT shell was reduced in the longitudinal direction and formed a wrinkled structure. In the stretching state, the wrinkled PVDF-TrFE/CNT shell was unwrinkled, resulting a constant of the electrical pathway of the CNTs. The thickness of electrospun PVDF-TrFE mats and CNT sheets were 30 um and 1 um, respectively (Supplementary Fig. S4). As a result, microdiameter STEF with an average diameter of 490 μm was fabricated using two types of stretchable electrodes, namely silver-coated nylon/PU fiber and PVDF-TrFE/CNT shell (Fig. 1e).

### Electrical energy generation process of stretchable triboelectric fiber

**Figure 2:**
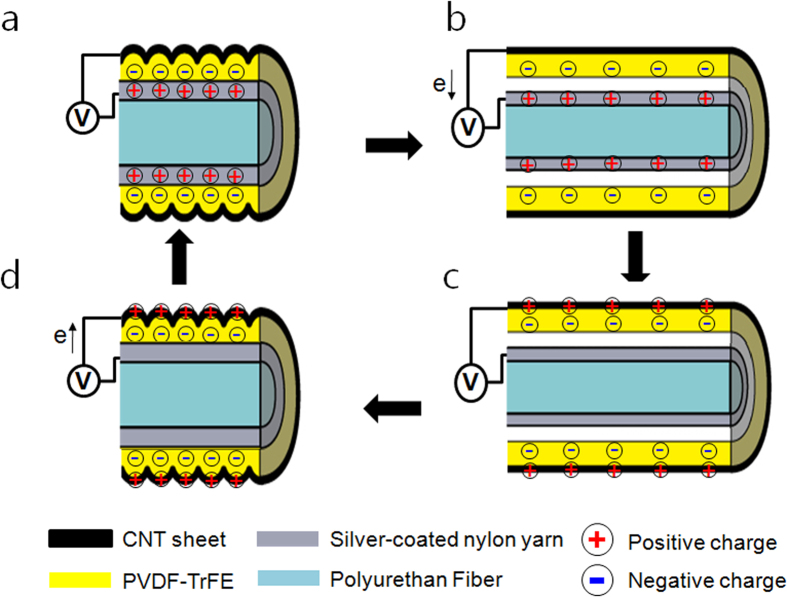
Electrical energy generation process of stretchable triboelectric fiber. (**a**) Initial state without applied strain; (**b**) the diameter of the silver-coated nylon/PU fiber decreases at high Poisson’s ratio of the PU fiber in stretching, causing separation between the silver-coated nylon/PU fiber and the PVDF-TrFE. The potential difference drives electrons from the CNT sheet to the silver-coated nylon; (**c**) the potential of the silver-coated nylon and CNT sheet reaches equilibrium; (**d**) the initial position is restored when the external force is removed. The electrons are driven back to the CNT sheet.

The electrical energy generation principle can be explained by the coupling between electrostatic and triboelectric effects^32^,^33^,^34^,^35^,^36^. At the initial state, the PVDF-TrFE mechanically contacts the silver-coated nylon. According to the triboelectric series, anion are bonded on the PVDF-TrFE surface by mechanical friction during the manufacturing process, resulting a generation of negative triboelectric charges on the PVDF-TrFE surface (Fig. 2a). When the fiber is stretched by external forces, the PVDF-TrFE and silver-coated nylon are separated because of the difference in Poisson’s ratio between the wrinkled PVDF-TrFE/CNT-shell and the silver-coated nylon/PU fiber (Fig. 2b). In the separated area between the PVDF- TrFE and the CNT, the CNT has a lower electric potential than the silver-coated nylon which produced a difference in electric potential by driving the electrons through the external loads. Eventually reaching equilibrium of electric potential (Fig. 2c). When the external force was removed, the STEF reversed to its initial position and the silver-coated nylon and PVDF-TrFE were brought into contact. As the PVDF-TrFE induced positive triboelectric charges on the silver-coated nylon, an electric potential difference between the CNT and the silver-coated nylon was generated. In consequence, the electrons flowed from the silver-coated nylon to the CNT (Fig. 2d) and kept screening the inductive charge until separation was again established (Fig. 2a).

When the silver-coated nylon/PU fiber was stretched, its diameter reduced from 440 μm at the initial state to 350 μm at a strain of 50% (Supplementary Fig. S5). However, when the STEF was stretched, the diameter changed from 490 μm at the initial state to 480 μm at a strain of 50% (Supplementary Fig. S6).

The Poisson’s ratio of a rod can be estimated from the equation^37^:


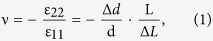


where ν is the Poisson’s ratio, ε_22_ is the lateral strain, ε_11_ is the axial strain, Δd is the changed diameter, d is the initial diameter, ΔL is the changed length, and L is the initial length of the fiber. According to eq. (1), the Poisson’s ratio of the silver-coated nylon/PU fiber and the STEF was 0.41 and 0.04, respectively. The Poisson’s ratio of the silver-coated nylon/PU fiber was thus 10 times greater than that of STEF, and the changed diameter ratio (Δd/d) of the silver-coated nylon/PU fiber was higher than that of the PVDF-TrFE/CNT shell. As the result, a free space was formed after stretching between the silver-coated nylon and the PVDF-TrFE.

To confirm the explanation above and the voltage response generated from the STEF, a switching polarity test was conducted (Supplementary Fig. S7). An open circuit voltage of 50 mm fiber was measured by an oscilloscope when the fiber was stretched by hand. With the forward electrical connection, a voltage pulse with the positive potential of the CNT and the negative potential of the silver-coated nylon was generated in the longitudinal direction by stretching. The potential difference between the CNT and the silver-coated nylon reached up to 240 mV when stretched and was later not observed when the STEF was hold at a strain of 50%. With the reversed electrical connection, the voltage pulse had the opposite tendency compared with the voltage pulse with forward connection; The short circuit problem is effectively prevented because the electrical insulating PVDF-TrFE mats are sandwiched between core and sheath electrodes. Although PVDF-TrFE is representative piezoelectric materials, piezoelectricity in the triboelectric fiber is negligible. The piezoelectricity of the PVDF-TrFE is only valid when the mats are contacted to top and bottom electrodes (when the dipole alignment is parallel to its thickness direction). However, in our case, the PVDF-TrFE mats lost its contact to core electrode in stretching state. Therefore, it can be concluded that not piezoelectricity but triboelectricity contributes to the power generation. Therefore, these results indicate that the voltage response was generated from the STEF, which supports the above explanation.

### Mechanical propertyandenergy generation performance of the stretchable triboelectric fiber

**Figure 3:**
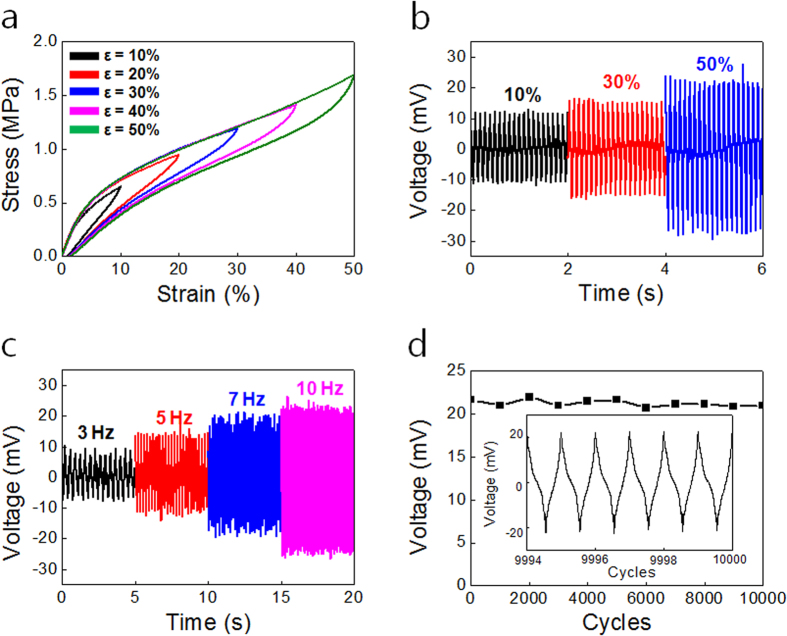
Mechanical property and energy generation performance of the stretchable triboelectric fiber. (**a**) Stress–strain curve of a triboelectric fiber after repeated stretching to different maximum strain from 10% to 50% and then released. The voltage response was measured for (**b**) varying strain ranging from 10% to 50% with a frequency of 10 Hz and (**c**) varying frequency from 3 Hz to 10 Hz at an applied strain of 50%. (**d**) The stability of the performance generated from the triboelectric fiber during 10,000 cycles to a maximum strain of 50% and at a 10 Hz frequency.

The elastic property of the STEF is shown in Fig. 3a. The stretch/release cycles showed the elastic recovery resulting in less than 1.68% residual deformation for strains up to 50%. The hysteresis between the stretching and releasing stress–strain curve represented a 26% energy loss for 50% strain.

To analyze the STEF performance with external stimuli, we experimentally used a linear motor to obtain an accurate performance with strain and frequency. At one end of the 3 mm fiber was fixed on the shelf and the other end was attached to the linear motor. The open circuit voltage and short circuit current was measured. The voltage response with applied strain ranging from 10% to 50% at a frequency of 10 Hz is shown in Fig. 3b. The generated voltage increased with strain from 13 mV at a strain of 10% strain to 24 mV at a strain of 50%. The current response with applied strain ranging from 10% to 50% at a frequency of 10 Hz was shown in Supplmentary Fig. S8a. The generated current increased with strain from 3 nA at strain of 10% to 8 nA at a strain of 50%. The integral transferred charges of the positive peak (Supplementary Fig. S9a) also showed an increase with strain from 5.5 pC at 10% to 10 pC at 50%. In general, the triboelectric generator performance increased with varying distance between the electrode and the triboelectric material^38^. When the distance between the electrode and surface of the triboelectric material increased, the number of inductive charges in the electrode decreased. As a result, the integral transferred charges and the triboelectric performance increased with increasing distance. According to eq. (1), there is a correlation between the distance silver-coated nylon and PVDF-TrFE and applied strain due to the Poisson’s ratio difference. When the maximum strain was applied, it showed the sensitivity of the triboelectric performance.

When we move in daily life, the frequency of human motion is below 10 Hz. To be able to detect human motion and to generate electrical energy from it, it was required to measure the triboelectric performance at low frequency below 10 Hz. The voltage output with varying frequency ranging from 3 to 10 Hz at a strain of 50% is presented in Fig. 3c. The voltage response increased with frequency from 9 mV at 3 Hz to 24 mV at 10 Hz at a strain of 50%. The current response with varying frequency ranging from 3 to 10 Hz at a strain of 50% was presented in Supplementary Fig. S8b. The current response increased with frequency from 2 nA at 3 Hz to 8 nA at 10 Hz at a strain of 50%. In general, the distance change between the silver-coated nylon and PVDF-TrFE is constant for a given applied strain and different applied frequency. The integral transferred charges of the positive peak were almost constant at 10 pC for different applied frequency (Supplementary Fig. S9b). Although the triboelectric performance increased with both strain and frequency, the strain and frequency could be measured by combining both the triboelectric performance and resistance change of the silver-coated nylon/PU fiber due to the resistance change which indicated the strain of the fiber (Supplementary Fig. S2a). When we measure both triboelectric performance and the resistance, the strain and frequency was determined using the voltage response graph with strain and frequency (Supplementary Fig. S10).

The stability of the STEF as a sensor is a critical issue for practical application. The STEF showed highly stable triboelectric performance during repeated deformation. An alternating voltage was generated for up to 10,000 stretching cycles at a strain of 50% and frequency of 10 Hz (Fig. 3d). The voltage response was constant during 10,000 cycles without distortion.

### Demonstration of kinematic sensing textile

**Figure 4:**
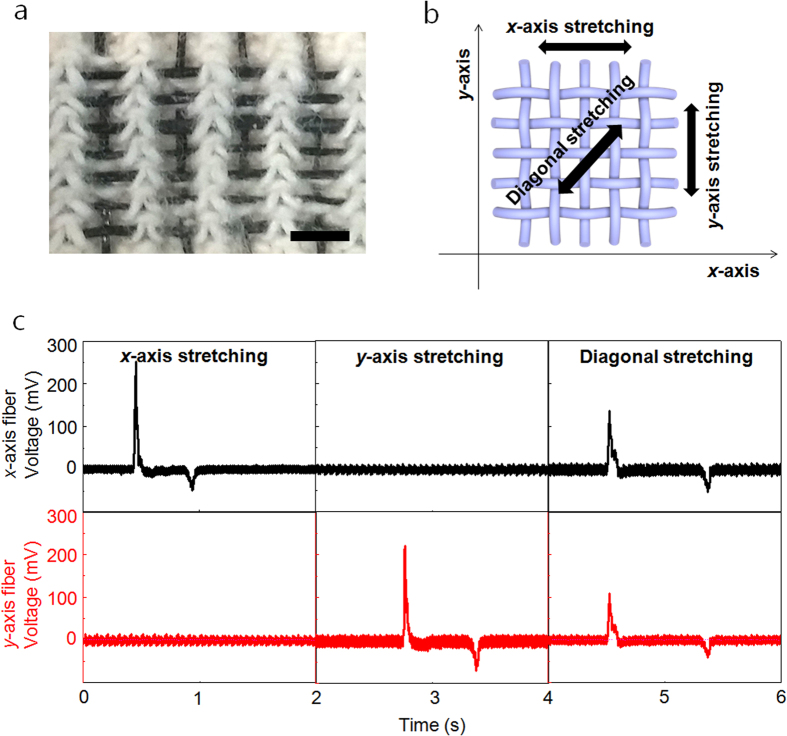
Demonstration of a kinematic sensing textile. (**a**) Optical image showing 50 mm triboelectric fibers woven into the wristband of a glove(scale bar: 3 mm). (**b**) Schematic diagram of the experimental setting and (**c**) voltage response of the *x*- and *y*-axes of the triboelectric fiber in a textile when strain was applied in the *x*-, *y*- and diagonal directions.

When human motion is measured, the information on the direction of motion is crucial for the monitoring system. The STEF suggested building block of application in kinematic sensing textile which can detect not only the magnitude but also its direction. To demonstrate its potential for textile processing, kinematic sensing textile was fabricated by plain weaving 11 individual fibers of 50 mm length (Fig. 4a). Each STEF was coated with elastomeric styrene-butylene-styrene to insulate the other fibers. When the textile was stretched in the *x*-, *y*-, and diagonal directions at a strain of 50% by hand, we measured the voltage response of the *x*- and *y*-axis fibers (Fig. 4b). When the textile was stretched in the *x*- or *y*-direction, the voltage response in each direction was detected from the respective fibers (Fig. 4c). Additionally, the voltage response from the *x*-axis fiber in stretching in the *x*-direction was higher than that in diagonal stretching as the strain in longitudinal stretching was higher than that in diagonal stretching. This kinematic sensing textile shows regular voltage response during not only stretching but also bending test (Supplementary Fig. S11). When the textile was bent and released, the voltage response detected from the respective fibers. This successfully demonstrated the accurate motion sensor, and it is possible to predict the direction of motion using two perpendicular triboelectric fibers, as these independently detect the perpendicular axis of the strain.

## Discussion

Stretchable triboelectric fiber was developed by wrapping a multishell triboelectric fiber—made by wrapping silver-coated nylon, electrospun PVDF-TrFE mats, and CNT sheet—around PU fiber. The Stretchable triboelectric fiber can be used as a strain sensor by measuring triboelectric performance and resistance change of the silver-coated nylon/PU fiber. The Stretchable triboelectric fiber showed good sensitivity and stability, and strain could be detected up to 50% at various frequencies up to 10 Hz. Furthermore, the kinematic sensing textile consisting of Stretchable triboelectric fiber could detect the direction of strain. This work demonstrates the new triboelectric mechanism of a self-powered strain sensor, and extends the application of fiber-based generators and smart textiles to detect human motion.

## Methods

### Sample fabrication of stretchable triboelectric fiber

To wrap the polyurethane (PU) fiber with silver-coated nylon (PN# 260151011717, 117/17 2-ply, StatexShieldex,USA), each end of the fiber was attached to a rotating motor. The 15 wt% PVDF-TrFE solution (70:30, Piezotech,France) was prepared by mixing 1.5 g PVDF-TrFE in 2.55 g dimethylacetamide (Sigma Aldrich, USA) and 5.95 g acetone (Sigma Aldrich, USA). The 10 wt% Styrene-butylene-styrene solution (Sigma Aldrich, USA) was prepared by mixing 1 g Styrene-butylene-styrene in 9 g chloroform (Sigma Aldrich, USA). The solutions were stirred for 24 h at room temperature. A voltage of 20 kV was applied between a syringe needle (15 kV) and a collector of aluminum foil (−5 kV) at a distance of 20 cm using high-voltage DC power supplies (Wookyong TECH, Korea). The polymer solutions were fed at a rate of 4 μl/min using a syringe pump (KD Scientific, USA). As-prepared electrospun PVDF-TrFE mats on the aluminum foil were cutina rectangular form of 1 cm × 10 cm. A silver-coated nylon/PU fiber was applied on the edge of the electrospun PVDF-TrFE mats and manually rolled. The CNT sheets were drawn from a CNT forest fabricated by the CVD method. To wrap the fiber with CNT sheets, each end of the fiber was attached to the rotating motor. The CNT sheet drawn from the CNT forest was attached onto the fiber with an angle of 45° between the fiber and the CNT sheet. When the motor was rotated in constant velocity, the overall PVDF-TrFE fiber was wrapped with CNT sheets.

### Structure characterization and triboelectric performance measurement

To show the STEF morphology, we used field-emission scanning electron microscopy (FESEM, Hitachi S4700, Japan) (at 15 kV). The electrical measurements were conducted using a digital multimeter(Model 187, Fluke Corporation, USA). The 10 mm silver-coated nylon/PU fiber and the PVDF-TrFE/CNT shell were attached to vernier calipers and the resistance was measured at intervals of 5% strain. The mechanical test was performed with a universal testing machine (UTM, INSTRON 5966, INSTRON, USA). The mechanical measurements were performed after several training runs and the initial force of 50 mN to measure the initial STEF length was established. The triboelectric performance was measured using an oscilloscope (MOS9104A, Agilent Technologies, USA). For the extension test of the STEF, a linear motor (TV50009/S503/BAA60, TIRA, Germany) was used to control the stroke and frequency.

## Additional Information

**How to cite this article**: Sim, H. J. *et al.* Stretchable Triboelectric Fiber for Self-powered Kinematic Sensing Textile. *Sci. Rep.* **6**, 35153; doi: 10.1038/srep35153 (2016).

## Supplementary Material

Supplementary Information
