# Supplementary material for: Stretchable Triboelectric Fiber for Self-powered Kinematic Sensing Textile
Source: Sci Rep. 2016 Oct 11;6:35153. doi: 10.1038/srep35153 (PMC5057101; doi:10.1038/srep35153)
Supplement: Supplementary Information [file srep35153-s1.pdf]

Supporting Information

Stretchable Triboelectric Fiber for Kinematic Sensing Textile

*By Hyeon Jun Sim, Changsoon Choi, Shi Hyeong Kim, Kang Min Kim, Chang Jun Lee, Youn Tae Kim, Xavier Lepró, Ray H. Baughman and Seon Jeong Kim**

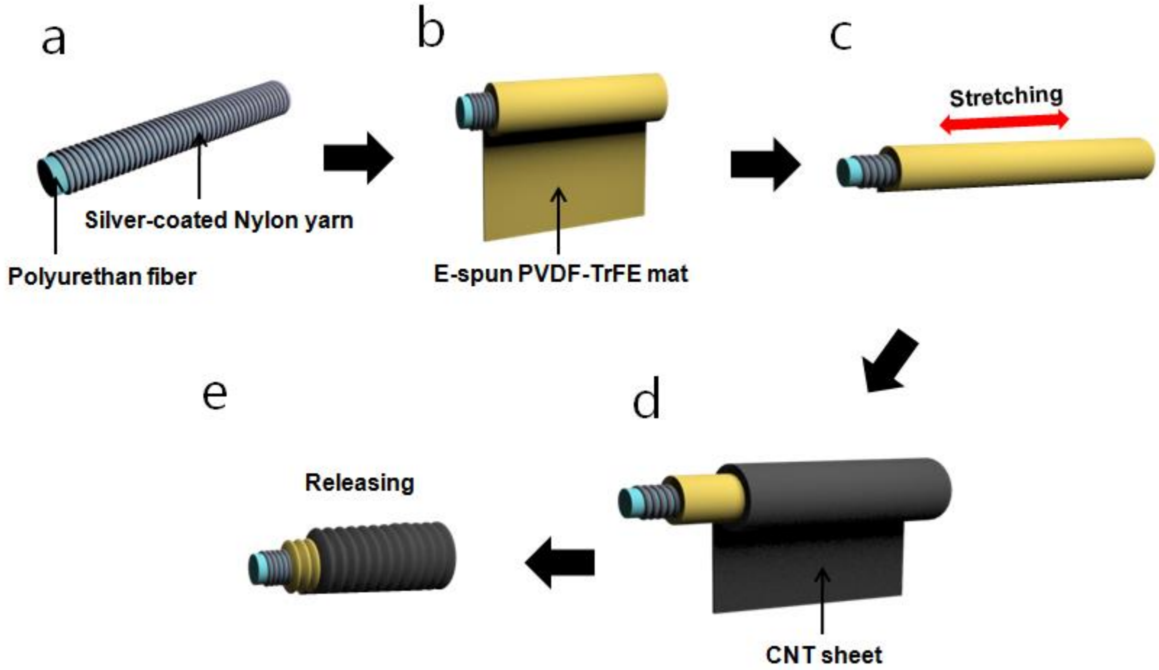

Figure S1. The fabrication process of the stretchable triboelectric fiber.

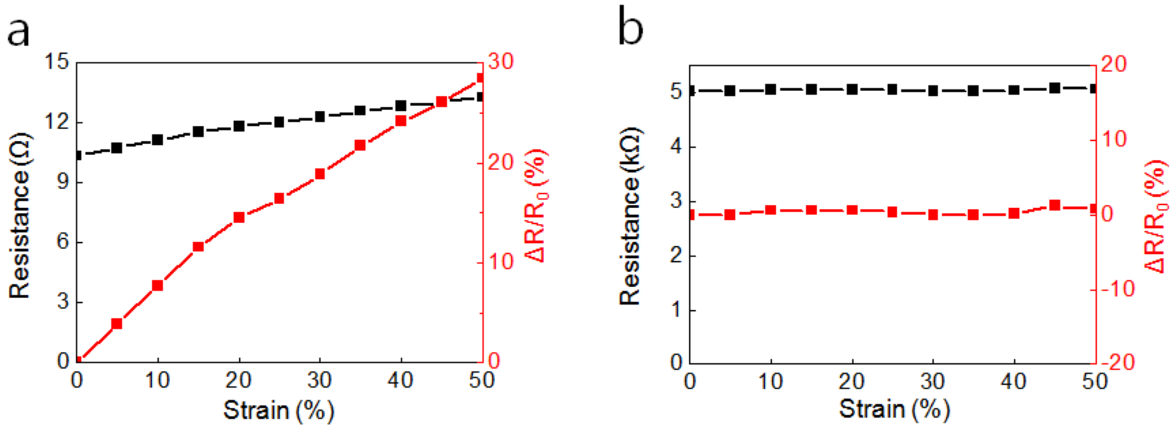

Figure S2. Resistance variation in longitudinal strain of **a,** 10 mm silver-coated nylon/PU fiber and **b,** 10 mm wrinkled PVDF-TrFE/CNT shell.

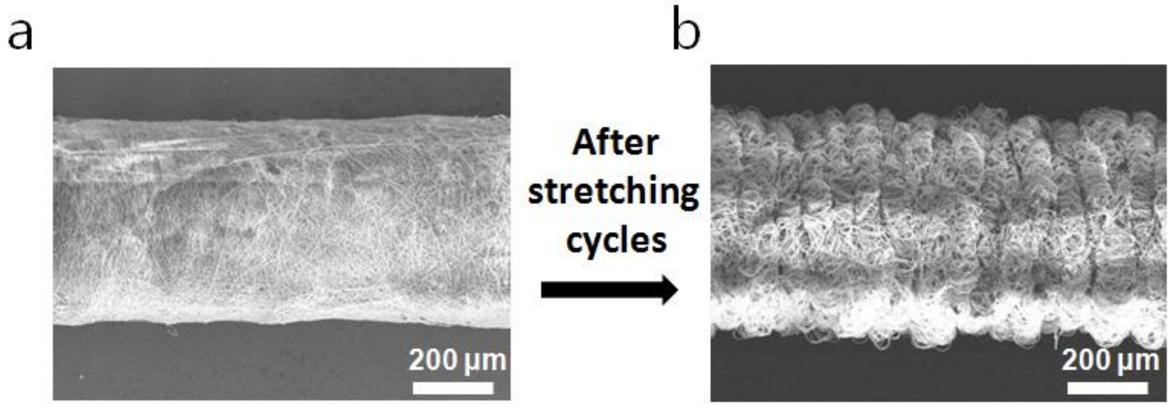

Figure S3. SEM image of the PVDF-TrFE/silver-coated nylon/PU fiber: **a,** initial state and **b,** after stretching cycles.

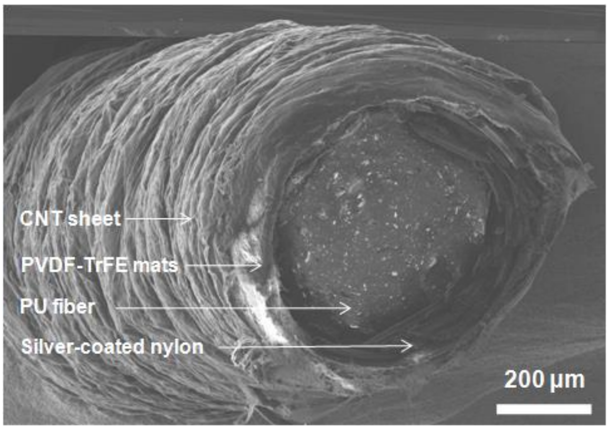

Figure S4. SEM image of the cross-section of stretchable triboelectric fiber

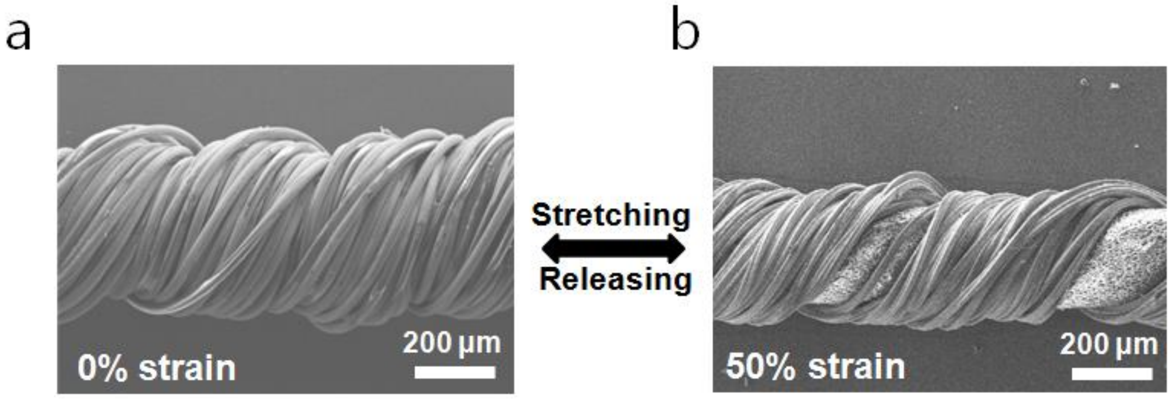

Figure S5. SEM image of the silver-coated nylon/PU fiber : **a,** initial state and **b,** stretching at 50% strain.

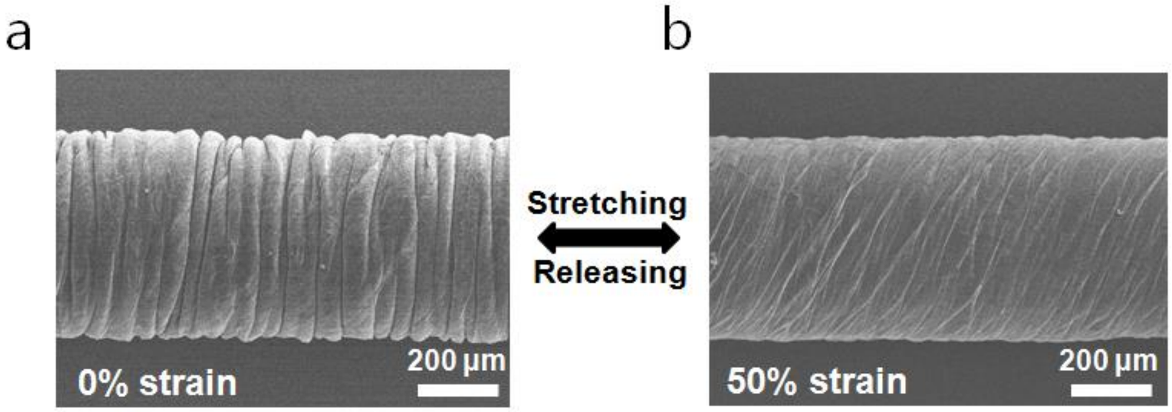

Figure S6. SEM image of the stretchable triboelectric fiber: **a,** initial state and **b,** stretching at 50% strain.

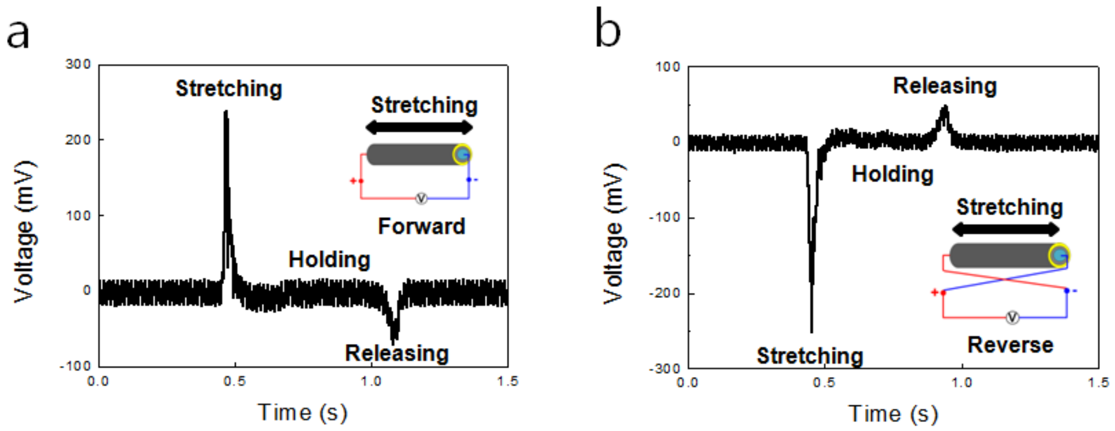

Figure S7. The open circuit voltage of 50 mm stretchable triboelectric fiber in **a,** forward connection and **b,** reversed connection during stretching by hand.

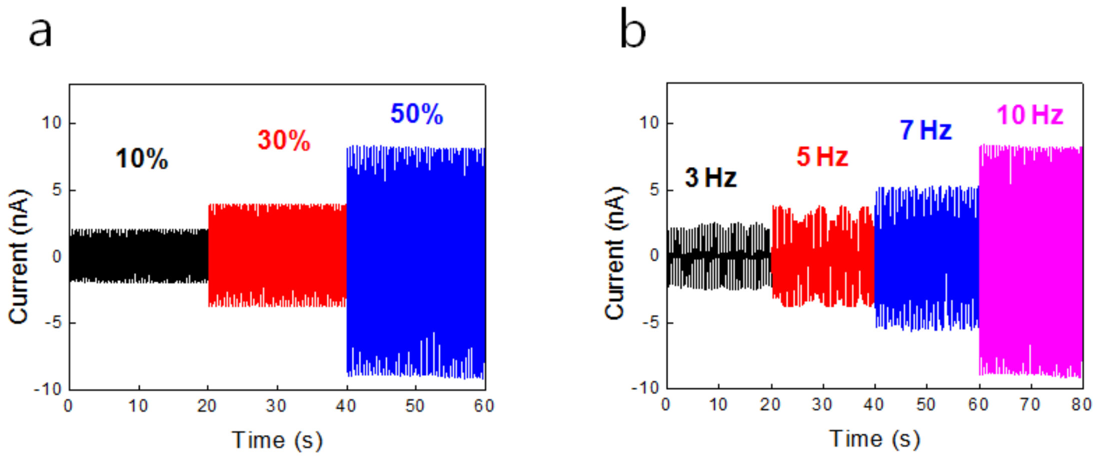

Figure S8. The current response was measured for **a,** varying strain ranging from 10% to 50% with a frequency of 10 Hz and **b,** varying frequency from 3 Hz to 10 Hz at an applied strain of 50%.

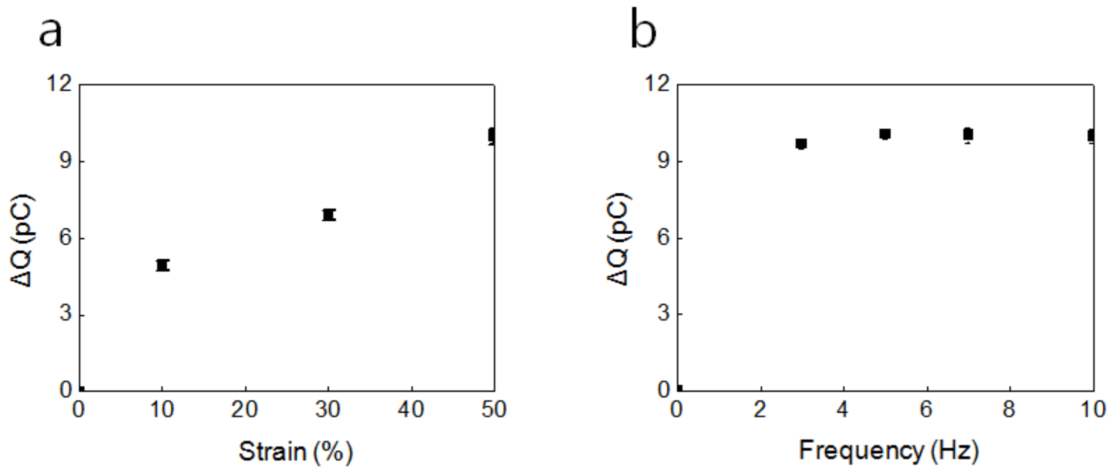

Figure S9. Variation in integral transferred charges of 3mm stretchable triboelectric fiber with **a,** applied strain of 10%, 30%, and 50% for a given frequency of 10 Hz and **b,** applied frequency of 3, 5, 7, and 10 Hz for a given strain of 50%.

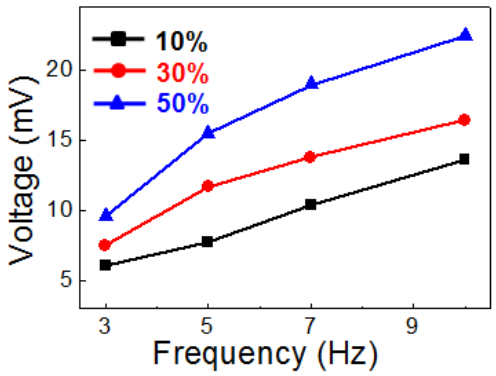

Figure S10. Voltage response of 3mm stretchable triboelectric fiber with different strains and frequencies.

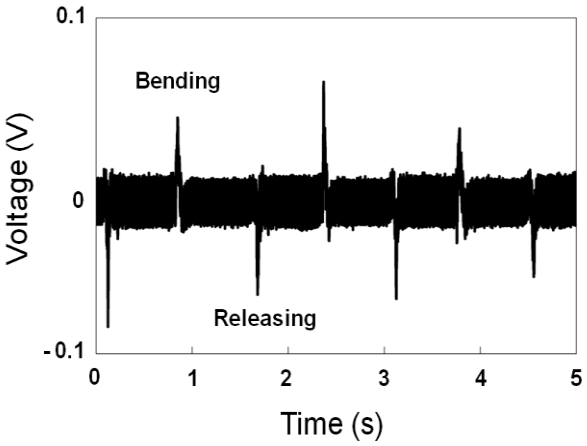

Figure S11. Current response of the triboelectric fiber in a textile when triboelectric fiber is in bending and releasing state.
